# Reversal of repetitive TMS-induced LTP-like plasticity by a novel compound in rats

**DOI:** 10.3389/fncir.2026.1925688

**Published:** 2026-09-08

**Authors:** Ugur Damar, Paul MacMullin, Sameer Dhamne, Alexander Rotenberg

**Affiliations:** F.M. Kirby Neurobiology Center, Neuromodulation Program, Department of Neurology, Boston Children’s Hospital, Harvard Medical School, Boston, MA, United States

**Keywords:** cortical plasticity, NMDAR, pentobarbital, repetitive TMS, SGE-301, metaplasticity

## Abstract

Repetitive transcranial magnetic stimulation (rTMS) modulates cortical plasticity in a frequency-dependent manner, resulting in predictable and lasting augmentation or attenuation of corticospinal excitability. These rTMS effects on synaptic plasticity are partly mediated by N-methyl-D-aspartate receptors (NMDARs) and share features with long-term depression (LTD) or long-term potentiation (LTP) of excitatory synaptic transmission. In this study, we investigated whether SGE 301 (an NMDAR positive allosteric modulator) alters rTMS-mediated motor cortical plasticity in pentobarbital-anesthetized rats. We tested two modalities: 1 Hz for the low-frequency rTMS (lf-rTMS) and 10 Hz for the high-frequency rTMS (hf-rTMS) to induce LTD-like and LTP-like plasticity, respectively. We compared corticospinal excitability before and after rTMS by measuring peak-to-peak motor evoked potential (MEP) amplitudes across a range of stimulator outputs. In the lf-rTMS experiment, the magnitude of the reduction of MEP amplitude was similar between the vehicle and SGE-301 groups. In the hf-rTMS experiment, however, the LTP-like plasticity observed in the vehicle group was completely reversed by SGE-301. In conclusion, SGE-301 engages cortical plasticity mechanisms *in vivo* and selectively prevents hf-rTMS-induced LTP-like plasticity without altering lf-rTMS-induced LTD-like plasticity. These findings support the utility of preclinical rTMS as a translational tool for detecting central target engagement by neuroplasticity-modulating compounds.

## 1 Introduction

Transcranial magnetic stimulation (TMS) is a non-invasive focal cortical stimulation method to measure or modulate cortical excitability through intracranial electrical currents induced by an extracranial fluctuating magnetic field. Specifically, when coupled with peripheral muscle readouts such as electromyogram (EMG) or mechanomyogram (MMG), TMS of the motor cortex can provide a range of target engagement biomarkers for pharmacotherapeutics that alter brain plasticity or excitability (Muller et al., 2014; O’Donnell et al., 2021).

Repetitive TMS (rTMS) protocols are designed to modify cortical excitability for clinical or experimental purposes. Two common rTMS protocols deliver prolonged trains of low-frequency (≤1 Hz) or high-frequency (≥5 Hz) stimulation to reduce or enhance regional cortical excitability, respectively. The mechanisms by which rTMS modifies cortical excitability remain incompletely understood; the frequency-dependent effects on cortical excitability and their differential responsiveness to inhibitors or positive modulators of the N-methyl-D-aspartate (NMDA)-type glutamate receptor (NMDAR) indicate a strong mechanistic overlap with use-dependent long-term depression (LTD) and long-term potentiation (LTP) of excitatory synaptic transmission that can be induced by the electrical stimulation of glutamatergic circuits (Edwards et al., 2008; Popovic and Dragic, 2025; Sohn et al., 2024). In this report, we assess the cortical plasticity induced by 1 Hz and 10 Hz rTMS and refer to these protocols as low-frequency rTMS (lf-rTMS) and high-frequency rTMS (hf-rTMS), respectively, in the sections below.

Modulators of NMDAR-mediated synaptic plasticity may have clinical utility. For instance, memantine, an NMDA-type glutamate receptor antagonist, is widely prescribed for memory enhancement in Alzheimer’s disease (Fox et al., 2025; Wang and Reddy, 2017). Amantadine, ketamine, and dextromethorphan are additional examples of clinically used NMDAR modulators: Amantadine acts as a low-affinity, uncompetitive NMDAR antagonist and is used for Parkinson’s disease and levodopa-induced dyskinesias; ketamine is a higher-affinity uncompetitive NMDAR open-channel blocker used for anesthesia, treatment-resistant depression, and chronic neuropathic pain; and dextromethorphan, also a low-affinity, uncompetitive NMDAR antagonist, is approved (in combination with quinidine) for pseudobulbar affect and is being investigated as an adjunctive therapy for epilepsy and depression (Kalia et al., 2008; Parsons et al., 1995; Zeng et al., 2025). Novel NMDAR modulators are also under investigation for autism spectrum disorder given that NMDAR dysfunction has been implicated in its pathophysiology (Deutsch and Burket, 2023; Lee et al., 2015). D-cycloserine, a partial agonist at the NMDAR glycine site; zelquistinel, an NMDAR positive allosteric modulator (PAM); and GluN2A-selective PAMs have shown promising results in animals and humans (Aye et al., 2021; Burket and Deutsch, 2019; Fonteneau et al., 2024; Modi and Young, 2011; Schade and Paulus, 2016). A recent addition to these established agents is SGE-301, a novel neuroactive steroid and PAM of NMDARs with preferential activity at GluN2A- and GluN2B-containing receptors, which is a synthetic analog of 24(S)-hydroxycholesterol and enhances synaptic plasticity by binding to a modulatory site on the NMDAR complex and prolonging channel open time, thereby potentiating NMDAR-mediated synaptic currents (La et al., 2019; Tang et al., 2023). SGE-301 restores NMDAR function in animal models of anti-NMDAR encephalitis and loss-of-function *GRIN* gene variants (Mannara et al., 2020; Tang et al., 2023). For compounds of this kind, a translational biomarker protocol to confirm central target engagement and functional modulation of cortical synaptic plasticity *in vivo* is highly desirable. rTMS is a well-suited tool for this purpose and is available in rodent models and humans (Caulfield et al., 2022; Pereira et al., 2016; Zong et al., 2022). We therefore used rTMS-induced motor cortical plasticity as a functional readout to (1) evaluate whether SGE-301 engages its CNS target *in vivo* and (2) determine whether SGE-301 differentially alters LTD-like versus LTP-like cortical plasticity.

## 2 Methods

### 2.1 Animals

Data from 48 adult male Sprague–Dawley rats (body weight: 280–420 g) were included in this set of experiments. Animals were housed in standard cages within a temperature- and humidity-controlled facility maintained on a 12-h light/dark cycle with continuous *ad libitum* access to food and water. All animal procedures were approved by the Institutional Animal Care and Use Committee (IACUC) at Boston Children’s Hospital and were conducted in accordance with the National Institutes of Health (NIH) Guide for the Care and Use of Laboratory Animals.

### 2.2 Compound and anesthesia administration

SGE-301 was obtained from Sage Therapeutics and formulated in 30% sulfobutylether β-cyclodextrin (SBECD, Captisol) at a concentration of 2 mg/mL. A single dose of SGE-301 (10 mg/kg) was administered intraperitoneally (i.p.) in all the experiments. An equal volume of 30% Captisol solution served as the vehicle. In the lf-rTMS experiments, pentobarbital was administered at two doses (65 mg/kg and 25 mg/kg, i.p.), 10 min apart, to achieve a stable level of anesthesia sufficient to inhibit voluntary movement and pain perception while preserving motor evoked potential (MEP) generation. In the hf-rTMS experiments, a single dose of pentobarbital (65 mg/kg, i.p.) was administered for anesthesia. Anesthesia was induced with 4% isoflurane in an induction chamber for 30–60 s, until the animal became immobile, and was followed immediately by pentobarbital injection.

### 2.3 Electrophysiological recordings

After pentobarbital administration, the desired depth of anesthesia was confirmed by the absence of the paw withdrawal reflex, and the rats were mounted in a stereotaxic frame (Stoelting Co., IL, USA). TMS was administered over the left motor cortex using a Magstim Rapid^2^ magnetic stimulator and a customized 70-mm figure-of-eight coil (40 mm external diameter for each lobe; Magstim Co., UK; Muller et al., 2014). The rTMS was set at the resting motor threshold (rMT), which is defined as the minimum machine output (MO) that generates a ≥ 0.2 mV peak-to-peak MEP amplitude in the contralateral brachioradialis muscle in at least 50% of consecutive pulses. For the sham rTMS groups, the same procedures were followed, except that stimulation was deployed at 5% MO.

The stimulation parameters were similar to the experimental setup previously described by our group (Muller et al., 2014; Rotenberg et al., 2010). The Rapid^2^ platform used in this study delivers biphasic pulses only. Regarding the remaining electrophysiological parameters, we note that the coil used in these experiments was a customized figure-of-eight coil, so the peak magnetic field and peak rate of change of current could not be taken from manufacturer specifications for stock coils, as both depend on specific coil inductance and geometry. These values were not measured directly in the present study, and the induced electric field and current density within the cortex were not computationally modeled. The stimulation site and coil orientation, however, were tightly controlled. The coil was mounted on a micromanipulator arm and rested in direct contact with the scalp, with the coil plane tangential to the skull at the target and the coil center approximately 9 mm lateral and 3 mm anterior to bregma. The coil was deliberately positioned eccentrically with respect to the scalp midline to allow lateralized MEPs in an animal whose head is considerably smaller than the stimulating coil.

Monopolar, uninsulated, 28G, 0.5″ stainless-steel needle electrodes were used to record EMG signals (Chalgren Enterprises Inc., Gilroy, CA, USA). Active recording electrodes were placed into the bilateral brachioradialis muscles to ensure strictly contralateral activation. Reference electrodes were placed subcutaneously between the third and fourth digits of the bilateral forepaws. The ground electrode was positioned subcutaneously on the dorsal surface of the tail. The EMG signal was bandpass-filtered from 100 to 1,000 Hz in the lf-rTMS experiments and from 300 to 20,000 Hz in the hf-rTMS experiments. The signals were amplified 1,000 × (A-M Systems Model 1700: Sequim, WA, USA) before digitalization at a sampling rate of 40 kHz (ADInstruments, Colorado Springs, CO, USA).

### 2.4 Statistical analyses

All statistical analyses were performed using GraphPad Prism (v 7.03; GraphPad Software Inc., La Jolla, CA, USA), with the statistical significance level defined as a *p*-value of < 0.05. *Post hoc* analyses were performed using Fisher’s LSD test. For graphical presentation, data are presented as mean ± SEM.

### 2.5 Lf-rTMS

A total of 19 rats were included in this set of experiments (rTMS-Vehicle = 5, rTMS-SGE-301 = 5, Sham–Vehicle = 5, and Sham–SGE-301 = 4). Fifty minutes after SGE-301 or vehicle administration, lf-rTMS was delivered at 1 Hz for 5 min (300 pulses). Optimal signal quality was elicited at 50–70% MO. For each stimulation intensity, 10 MEP amplitudes evoked at 7-s intervals were averaged to generate pre-rTMS, post-rTMS I, and post-rTMS II recruitment curves (Figure 1A).

**Figure 1:**
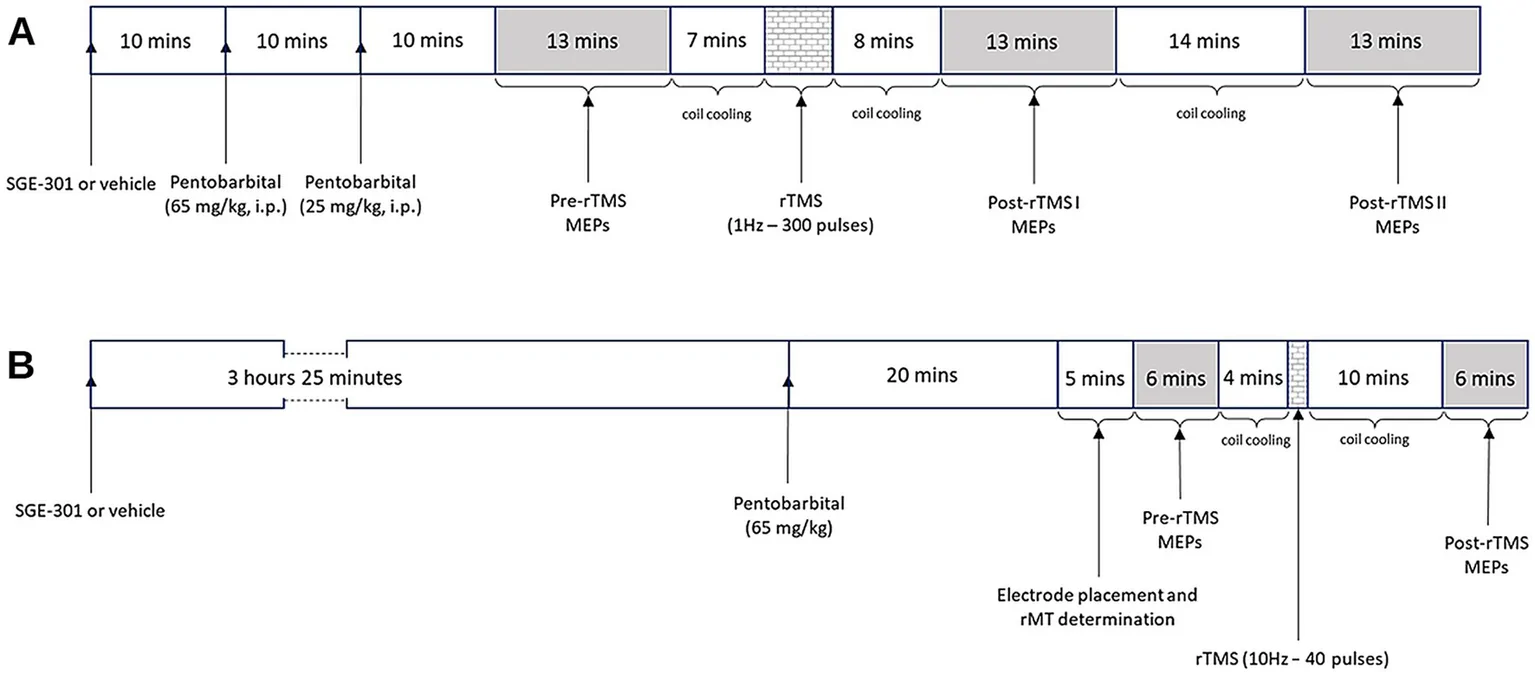
rTMS experiments. Recruitment curves from solid grey boxes were used for data analysis. **(A)** lf-rTMS experimental protocol. Vehicle or SGE-301 administration was 50 min before 1-Hz rTMS. **(B)** hf-rTMS experimental protocol. Vehicle or SGE-301 administration was 4 h before 10-Hz rTMS. Timelines reflect distinct train length and technical (e.g., coil cooling) requirements of lf-rTMS and hf-rTMS protocols.

The median increase in MEP amplitude at each intensity resulting from pentobarbital washout in the sham lf-rTMS arms was added to the corresponding pre-rTMS mean MEP values in the active (verum) lf-rTMS arms (Figure 2). The original pre-rTMS recruitment curves in the active lf-rTMS groups were thus replaced with these sham-adjusted pre-rTMS recruitment curves at each post-rTMS time point for comparison across post-rTMS time points (Figures 3A–D).

**Figure 2:**
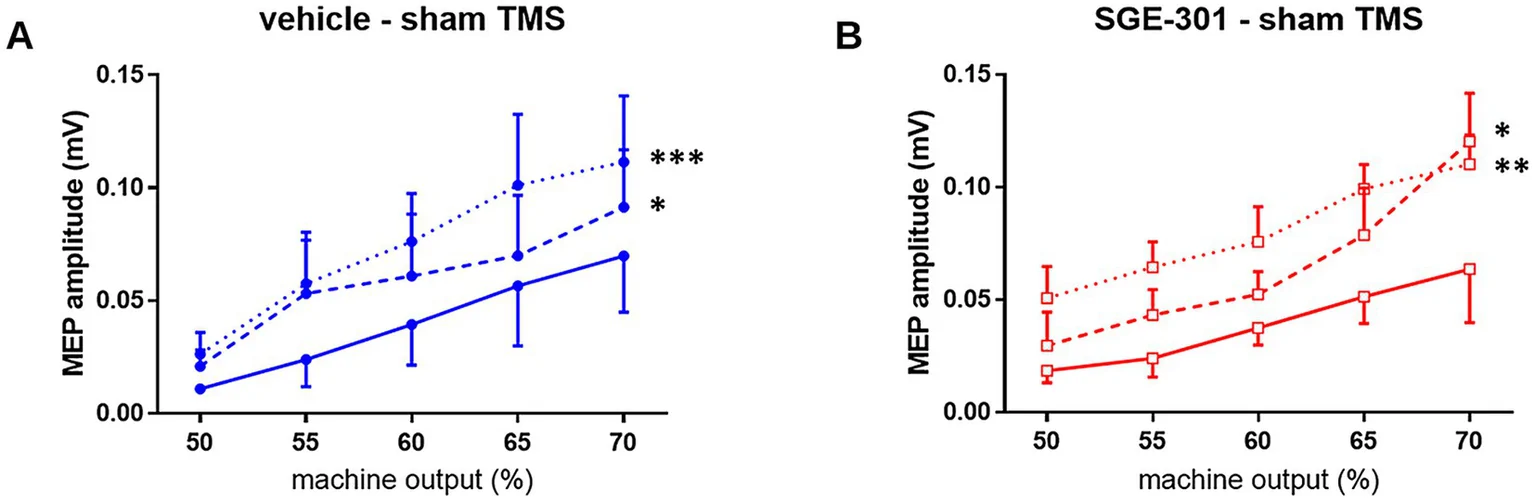
Pentobarbital washout in sham-rTMS groups. MEP amplitudes were higher after sham rTMS than those before rTMS due to reduced depth of anesthesia in the vehicle (blue) **(A)** and SGE-301 (red) **(B)** groups. The magnitude of increase between the two time points after rTMS was not different in either group (solid, dashed, and dotted lines represent pre-TMS, post-rTMS I, and post-rTMS II curves, respectively; *p < 0.05, **p < 0.01, ***p < 0.001).

**Figure 3:**
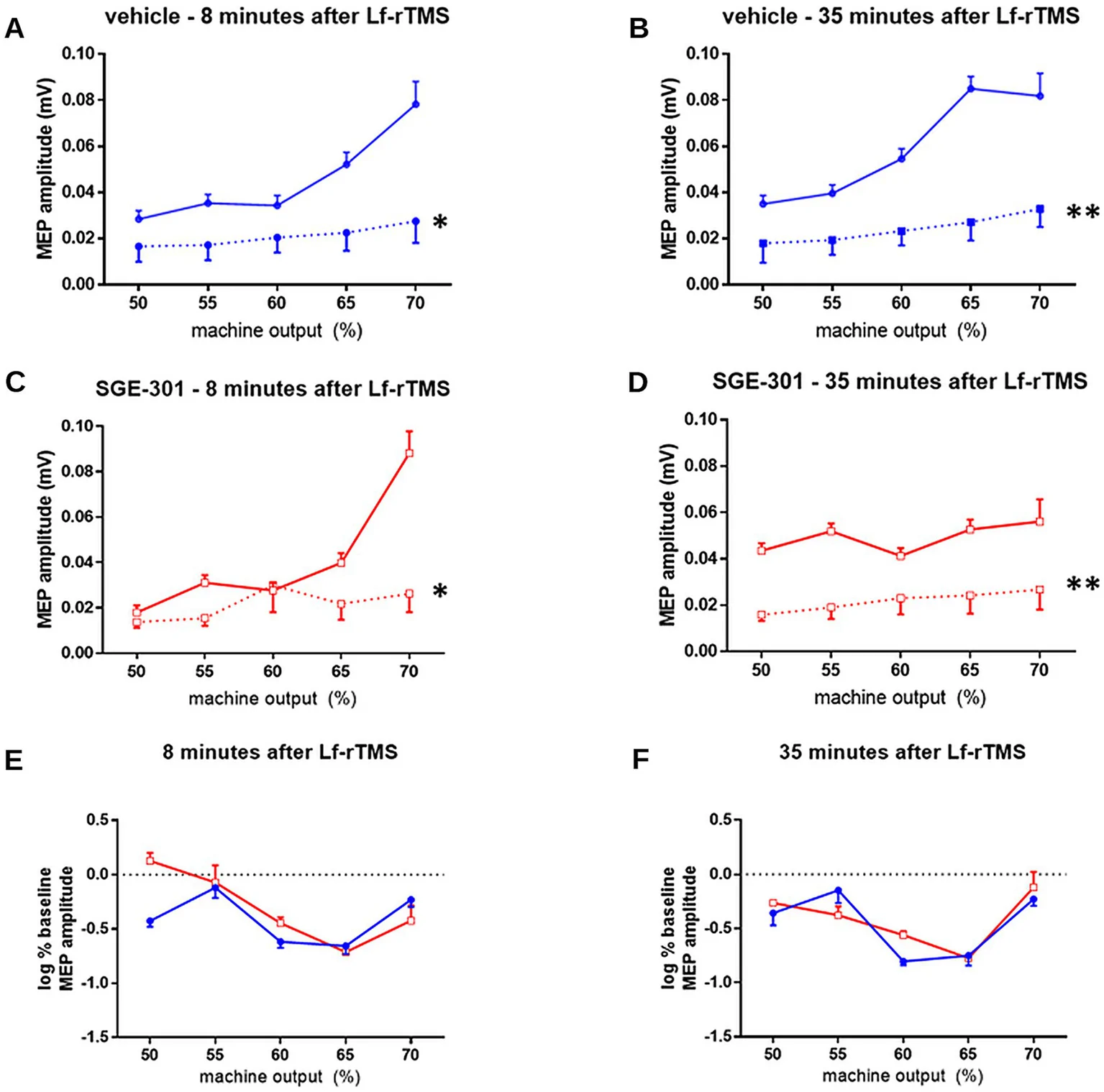
LTD-like plasticity 8 and 35 min after LF-rTMS. LF-rTMS-mediated MEP amplitude reduction after vehicle (blue) **(A,B)** and SGE-301 (red) **(C,D)** groups. (solid and dotted lines represent pre- and post-rTMS curves, respectively; *p < 0.05, **p < 0.01, ***p < 0.001). Magnitude of MEP amplitude reduction was similar **(E,F)** between groups (closed circles and hollow squares represent vehicle and SGE-301 groups, respectively).

### 2.6 Hf-rTMS

A total of 29 rats were included in this set of experiments (rTMS-SGE-301 = 15 and rTMS-Vehicle = 14). SGE-301 effects were assessed 4 h after administration, a time point at which brain concentrations of the compound remained appreciable (data not shown). hf-rTMS was applied at 10 Hz for 4 s (40 pulses). Optimal signal quality was elicited at 65–85% MO. Recruitment curves were generated in the same way as in lf-rTMS experiments, except for a single post-rTMS data point, which was 10 min after rTMS (Figure 1B). The 10 Hz frequency and the 4 s train duration were selected based on prior studies, including the work from our own group (Michel-Flutot et al., 2021; Vahabzadeh-Hagh et al., 2012). The figure-of-eight coil used in this study has a limited capacity to dissipate heat at high stimulation frequencies; therefore, this experimental configuration required an active cooling interval before the coil could be functional again (Muller et al., 2014).

## 3 Results

### 3.1 Lf-rTMS produced LTD-like plasticity in the vehicle and SGE-301 groups

A two-way ANOVA with repeated measures of MO and group as factors showed increased MEP amplitudes in both the vehicle (*p* = 0.002) and SGE-301 (*p* = 0.010) groups at 8 and 35 min after sham lf-rTMS (Figure 2). In neither group was there a significant difference between the post-rTMS I and post-rTMS II curves. After accounting for pentobarbital washout and generating sham-adjusted pre-rTMS curves, a two-way ANOVA with repeated measures of MO and group as factors showed that the MEP amplitudes were significantly reduced in the vehicle (*p* = 0.013 and *p* = 0.006, respectively) and SGE-301 (*p* = 0.028 and *p* = 0.011, respectively) groups, at 8 and 35 min after lf-rTMS, respectively (Figures 3A–D). However, the magnitude of MEP amplitude reduction in the SGE-301 group was not greater than that in the vehicle group (Figures 3E,F). After accounting for pentobarbital washout, all animals in both active lf-rTMS groups showed reduced MEP amplitudes relative to the sham-adjusted baseline, consistent with the group-level LTD-like effect.

### 3.2 Hf-rTMS-induced LTP-like plasticity was reversed by SGE-301

A two-way ANOVA with repeated measures of both MO and group as factors showed that MEP amplitudes were significantly increased in the vehicle group (*p* = 0.042) and decreased in the SGE-301 group (*p* = 0.048) after 10-Hz rTMS (Figures 4A,B). Moreover, a two-way ANOVA with repeated measures of only MO showed that log percent baseline MEP amplitudes in the SGE-301 group were lower than those in the vehicle group (*p* = 0.004) after hf-rTMS (Figure 4C). The average MEP amplitude change from baseline per animal was also significantly lower in the SGE-301 group than in the vehicle group (*p* = 0.004). Pre-rTMS recruitment curves, which were elicited 3 h 50 min after vehicle or SGE-301 administration, were not different. Individual responses to hf-rTMS were more heterogeneous and varied with stimulation intensity. Across intensities, 6–7 of the 15 animals in the SGE-301 group showed increased MEP amplitudes relative to baseline, with the remainder showing a reduction consistent with a reversal of the expected LTP-like facilitation, compared with 8–13 of the 14 animals in the vehicle group that showed persistent facilitation.

**Figure 4:**
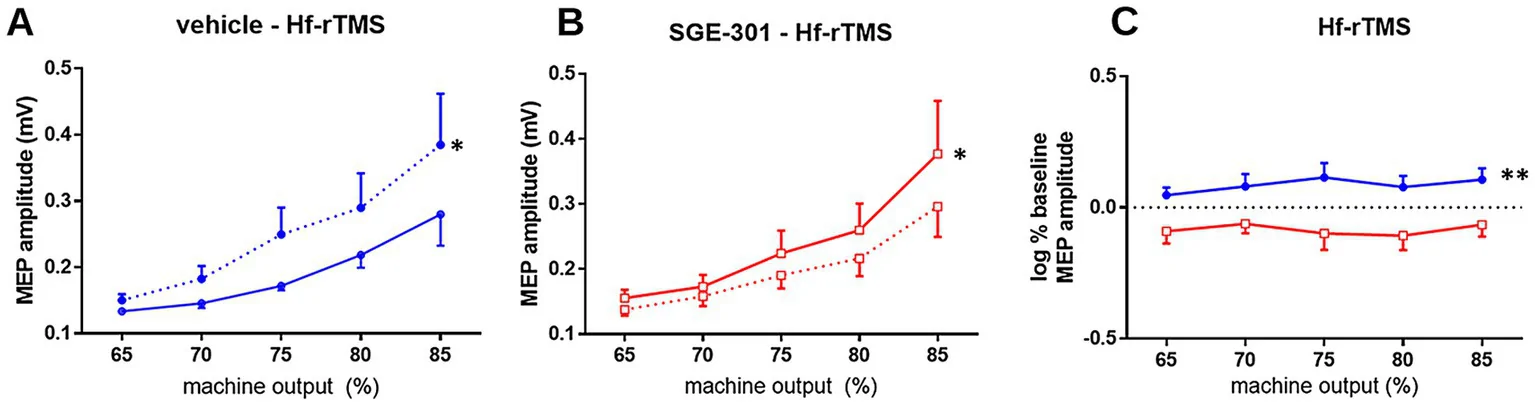
LTP-like plasticity after hf-rTMS. **(A)** MEP amplitude augmentation after hf-rTMS and vehicle. **(B)** MEP amplitude reduction after hf-rTMS and SGE-301. **(C)** Opposite changes in MEP amplitudes after hf-rTMS with vehicle or SGE-301 (solid and dotted lines represent pre- and post-rTMS curves, respectively. Closed circles and hollow squares represent vehicle (blue) and SGE-301 (red) groups, respectively. *p < 0.05, **p < 0.01).

## 4 Discussion

rTMS has been proposed as a translational tool for quantifying central target engagement by drugs that affect plasticity. In animal and *in vitro* models, rTMS can induce LTP- and LTD-like plasticity, facilitating mechanistic studies of how novel compounds influence plasticity pathways, including NMDAR-dependent processes (Popovic and Dragic, 2025; Tang et al., 2017). Clinically, rTMS protocols can be paired with pharmacological agents to assess whether candidate drugs enhance synaptic plasticity or rescue plasticity deficits. For instance, D-cycloserine, a partial NMDA receptor agonist, when used as an adjunct with rTMS, led to greater improvements in depressive symptoms and higher rates of clinical response, supporting the use of rTMS as a functional assay for drug-induced plasticity enhancement (Cole et al., 2022; Wrightson et al., 2023). Consequently, rTMS can provide a non-invasive readout of synaptic plasticity in both humans and rodent models, potentially bridging mechanistic insights from preclinical models to clinical trials and enabling the objective evaluation of central drug effects.

In this study, we found that SGE-301, a positive allosteric modulator of NMDARs, did not enhance lf-rTMS-induced LTD-like plasticity, but instead selectively reversed hf-rTMS-induced LTP-like plasticity. The absence of an augmented LTD-like effect in the lf-rTMS condition may reflect a floor effect, as lf-rTMS alone produced a pronounced reduction in corticospinal excitability. In contrast, the paradoxical reduction in MEP amplitudes following hf-rTMS in the SGE-301 group may suggest a metaplastic shift in the thresholds governing cortical excitability plasticity, in which baseline enhancement of NMDAR signaling lowers the threshold for synaptic depression, thereby rendering the subsequent stimulation more likely to produce LTD-like outcomes as a homeostatic mechanism (Abbott and Nelson, 2000; Abraham and Bear, 1996; Karabanov et al., 2015). This interpretation is consistent with prior evidence that SGE-301 restores impaired LTP in a mouse model of anti-NMDAR encephalitis (Mannara et al., 2020). Moreover, SGE-301 has been reported to enhance both GluN2A- and GluN2B-containing NMDAR activity, with these subunits in turn being linked to the regulation of the LTP–LTD threshold (Kostakis et al., 2013; Paoletti et al., 2013; Tang et al., 2023). These converging lines of evidence suggest that the impact of SGE-301 on cortical plasticity may involve complex homeostatic regulation of NMDAR-mediated synaptic transmission, a hypothesis that warrants further investigation. Taken together, these data are consistent with the use of TMS as a translational tool that could detect target engagement by centrally acting compounds and help guide subsequent steps in understanding the mechanisms of their potential therapeutic effects.

Metaplasticity is not the only plausible explanation for the divergent effects of SGE-301, and several alternative mechanisms merit consideration. First, the rTMS effects reflect the net activity of excitatory and inhibitory circuits, and high- and low-frequency protocols may engage cortical inhibitory interneurons differently. A PAM acting on NMDARs expressed on interneurons could shift this excitation–inhibition balance rather than acting solely at excitatory synapses (Benali et al., 2011; Kweon et al., 2026). It is important to note that this possibility is compounded in this study given the positive allosteric modulatory effects of pentobarbital at GABA-A receptors. Second, the two experiments differed by approximately 3 h in the interval between dosing and stimulation. Therefore, at least part of the difference could be attributed to SGE-301 pharmacokinetic differences. Third, downstream processes, such as AMPA receptor trafficking and BDNF signaling, are differentially affected by high- versus low-frequency rTMS, which could contribute to the group difference (Gersner et al., 2011). Finally, none of these mechanisms fully accounts for the within-group variability we observed. Such variability is consistent with findings from the human and animal rTMS literature, though its physiological basis remains unclear. This suggests that describing lf-rTMS as inhibitory and hf-rTMS as facilitatory is best considered a group-level tendency rather than a fixed property of these protocols (Maeda et al., 2000; Magnuson et al., 2023).

We employed an anesthesia protocol in the lf-rTMS experiments where we aimed to sustain anesthesia depth by two successive pentobarbital doses, enabling prolonged EMG recordings similar to protocols previously described in our laboratory (Muller et al., 2014). We observed a gradual increase in MEP amplitudes in the sham-rTMS groups, suggesting pentobarbital washout over the course of the experiment (Figure 2). To delineate lf-rTMS effects while accounting for this gradual weaning of anesthesia, we generated sham-adjusted pre-rTMS recruitment curves for each animal in the active (verum) rTMS groups. This method could provide more consistent results in animal TMS studies that necessitate multiple longitudinal time points.

There are several limitations to our study. One is the difference in the timing of stimulation between the low- and high-frequency rTMS experiments, with the latter involving approximately a 3-h longer period between SGE-301 administration and TMS. Therefore, it is difficult to directly compare the effects of SGE-301 on LTD- and LTP-like plasticity. Another limitation is that the sample size in the lf-rTMS experiment was smaller than that in the hf-rTMS experiment. Further studies with larger sample sizes could reveal differential SGE-301 effects on distinct plasticity mechanisms. It is worthwhile to note that the TMS coil used in this study is substantially larger than the rat head, and spatial focality is inherently limited relative to human TMS. Therefore, changes in MEP amplitude cannot be attributed exclusively to the primary motor cortex.

In summary, SGE-301, an NMDAR PAM, selectively reversed hf-rTMS-induced LTP-like plasticity in the rat motor cortex while leaving lf-rTMS-induced LTD-like plasticity essentially unchanged. The reversal of the expected direction of response to hf-rTMS demonstrates that SGE-301 modulated cortical plasticity mechanisms *in vivo*, consistent with—though not proof of—a homeostatic metaplastic shift in the threshold between synaptic potentiation and depression. These findings support the use of preclinical rTMS as a translational biomarker tool for detecting central target engagement by plasticity-modulating compounds, while the precise mechanistic basis underlying the divergent effects across stimulation protocols warrants further investigation.

## Data Availability

The raw data supporting the conclusions of this article will be made available by the authors, without undue reservation.
